# A randomized clinical trial comparing Hall vs conventional technique in placing preformed metal crowns from Sudan

**DOI:** 10.1371/journal.pone.0217740

**Published:** 2019-06-03

**Authors:** Fadil Elamin, Nihal Abdelazeem, Isra Salah, Yousra Mirghani, Ferranti Wong

**Affiliations:** 1 Queen Mary University of London, Institute of Dentistry, Bart's and The London School of Medicine and Dentistry, London, United Kingdom; 2 Khartoum Centre for Research and Medical Training, Khartoum, Sudan; University of Melbourne, AUSTRALIA

## Abstract

Despite the high success rates of preformed metal crowns (PMCs) in children no randomized clinical trials compare methods of placement and none describe its use in Africa. Our aim was to compare survival and cost-effectiveness of PMCs placed by conventional techniques (CT) and biological Hall techniques (HT) using a prospective randomized control trial in a general dental practice from Khartoum. One hundred and nine and 103 PMCs were placed in randomly selected children (5-8years) with 1–2 carious primary molars using HT and CT respectively and followed for 2 years. Socioeconomic status, periodontal health, occlusion, anxiety, and procedure time were compared using student t-test. Kaplan–Meier survival rates and incremental cost effectiveness ratio (ICER) were compared between CT and HT. CT and HT groups were similar for age, gender, socio-economic status. Survival rates were high (over 90%) for both study arms and not statistically different (p>0.05). Anxiety scores were significantly higher in CT arm after 12 months compared to HT (p<0.001). Clinically, gingival and plaque indices were similar between groups (p>0.05) but occlusions were raised in nearly all subjects in the HT arm (p<0.05). Periodontal health improved, and occlusions adjusted over time in both arms. There were 3 (2.7%) and 6 (5.8%) minor failures, 7 (6.4%) and 6 (5.8%) major failures in HT and CT arms respectively. Mean procedure time was lower in HT (9.1 min) than CT (33.9 min); p<0.001. Mean PMC cost was US$2.45 and US$7.81 for HT and CT respectively. The ICER was US$136.56 more for each PMC placed by CT per life year. We show that PMCs have high survival outcomes in disadvantaged populations similar to results from developed countries. As HT can be carried out by less experienced dental operators and therapists, this biological approach provides a promising cost-effective option to manage caries in developing countries with limited resources.

**Trial registration:** The trial is registered at clinicaltrials.gov. ClinicalTrial.gov Trial Registration: NCT03640013

## Introduction

Preformed metal crowns (PMCs) have excellent and higher success rates for treating carious primary molars in children compared to conventional restorations such as composite resin restorations, glass ionomer cements and amalgam [1–6]. They are routinely used in specialist paediatric practices in developed countries but relatively underutilised by general practitioners worldwide [6–10]. Restoration of carious molars is carried out using the conventional technique which involves total carious tissue removal. As the CT requires tooth modification and occlusal reduction, it usually requires the administration of local anaesthetic and rubber dam use [11,12]. Hence, it is regarded by general dental practitioners to be highly technical, requiring advanced training, including excellent child behaviour management skills. It is therefore highly unlikely to be widely used outside specialist settings and unlikely to be incorporated in routine care of deprived children from developing countries. The Hall technique (HT) on the other hand, requires either no, or partial, carious tissue removal, and no tooth modification or local anaesthesia. The PMC is cemented by the clinician using a gentle push aided by the child’s biting force. This biological approach has been shown to have high success rate [6].

Despite the fact that HT uptake is increasing amongst educational institutions as well as specialists from developed countries [6], PMC use is yet to be explored in developing countries. Only two retrospective studies were found comparing the two techniques, showing similar success rates [13,14]. Direct comparison of the results between HT and CT from these studies is difficult as different methods were used.

To date, no published prospective randomized controlled study has compared the efficacy of CT and HT in placing PMCs. Hence, the aims of this study were to compare the clinical performance, success rate and cost-effectiveness of PMCs placed by either the HT or CT using a prospective randomized clinical trial design in Sudan.

## Materials and methods

The study was conducted in a general dental practice in Khartoum, Sudan (2014–2017). The practice has a throughput of 6000 to 6500 children per annum. Ethical approval was granted by the Khartoum Centre for Research and Medical Training Review Board on 11/9/2014 (Ref. Paed/Dent/11). Due to a clerical and managerial oversight, the trial was registered after enrolment of participants had started. This led to the retrospective inclusion of the study in the trials register (Clinicaltrials.gov; identifier NCT03640013). Recruitment began in February 2015 and ended in March 2016. The protocol has been submitted with the manuscript as supporting information. The authors confirm that all ongoing and related trials for this drug/intervention are registered. All relevant data are within the paper. The trial ended in May 2018.

### Sample size

A sample size of 146 teeth was needed to detect a significant difference using 80% power and α = 0.05 (two-sided) [15,16], based on previously reported success rates of 80% and 95% for both CT (occlusal and approximal) and HT SSCs [3,17–20]. Assuming a dropout rate of 20% the size required the inclusion of 230 teeth.

### Inclusion criteria

The children from the practice pool were scanned and those who were between 5 to 8 years old and medically fit were selected. From clinical and radiographic examination (using rotational tomograms, OPG, as the practice norm), those children who were diagnosed to have untreated single surface (occlusal) or multi-surface (approximal) cavitated carious lesion extending to dentine; i.e. ICDAS (4 and 5) and with or without marginal ridge breakdown, in 1 or 2 primary molars were invited and recruited in the study. Caries diagnosis and treatment planning for all participants were carried out by a single senior dentist from the clinic (FE). Written consents were obtained from the accompanying parents.

### Exclusion criteria

Children were excluded if they:

- Had chronic medical conditions requiring long-term specialist care e.g. immunocompromised, cardiovascular and bleeding disorders
- Were uncooperative during clinical examination
- Lived in remote villages and were unlikely to return for follow-up
- Had teeth with pain or sepsis
- Had teeth with carious lesion extending into pulp clinically or on radiographic examinations (if available). All teeth with approximal caries were radiographed. No radiographs were taken for lesions limited to occlusal surfaces.

### Trial design

**Fig 1:**
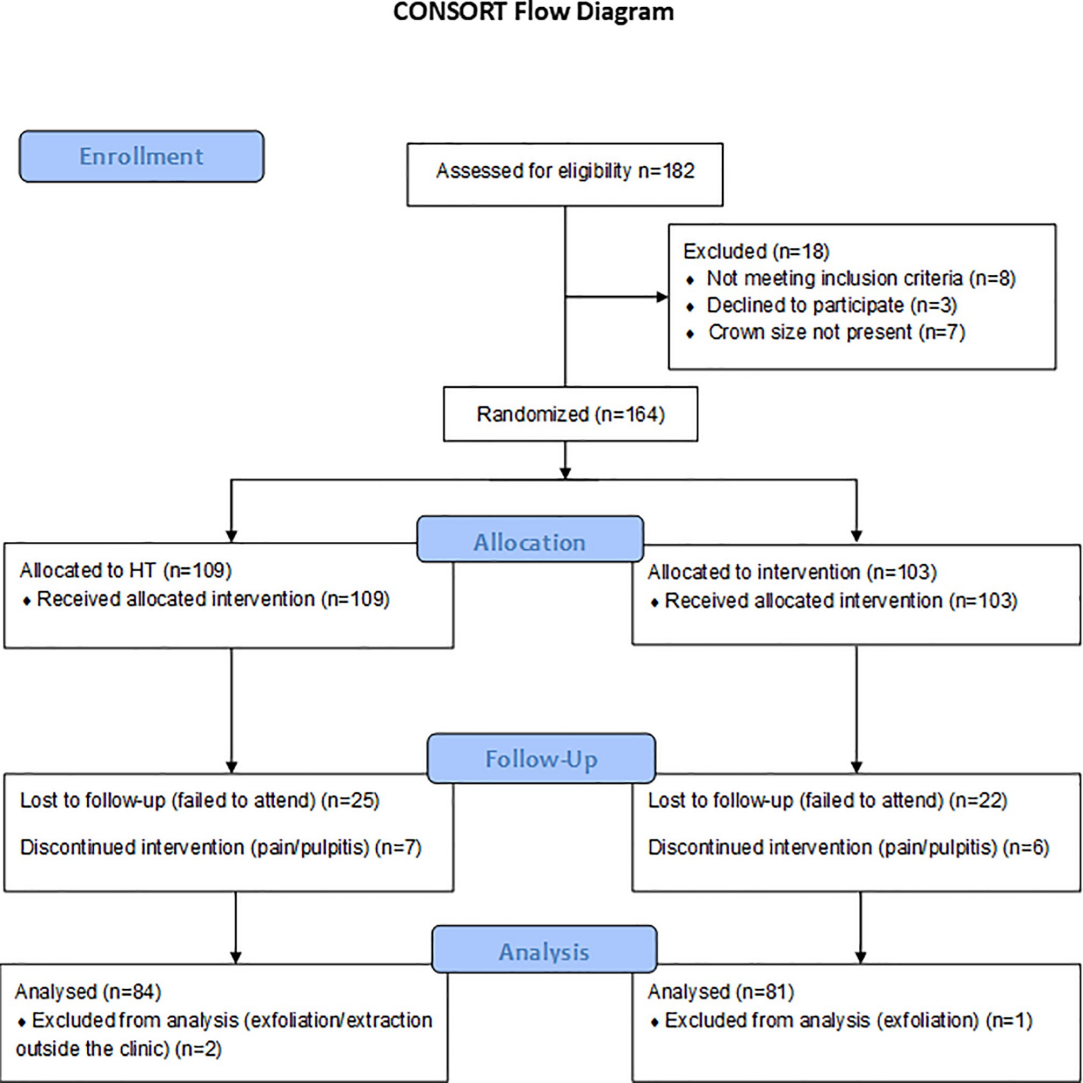
CONSORT flow diagram.

This prospective randomized clinical trial followed the CONSORT recommendations (Fig 1) [21]. 164 children fulfilled the inclusion criteria and were selected for the study. After the clinical data were recorded, the children were randomized by a dental assistant by tossing a coin and allocated to one of the two arms. The participants and the clinician (FE) assessing outcomes were blinded to the intervention provided.

1. **HT**–The PMCs were placed on the affected teeth (n = 109) in 86 patients using the published technique [17]. They were placed without local anaesthetic and without crimping or trimming. When the contact points were tight, an orthodontic elastic separator was placed for 2 hours prior to PMC placement (n = 7 teeth, 6.4%). The PMCs were cemented with glass ionomer (Vitrofil DFL, Brazil) at a powder/liquid ratio recommended by the manufacturer at room temperature. The HT procedures were carried out by a dental therapist with 2 years of experience after graduation (SI).
2. **CT**—Conventional preparation and reduction of the affected primary molars (n = 103 in 78 patients) were carried out [5] with local anaesthesia. The PMC were trimmed and crimped at the gingival margin to improve the fit and achieve occlusal contacts [11]. They were then adapted and cemented with the same type of glass ionomer as in the HT group with no rubber dam. All the procedures in this group were carried out by a dentist with an experience of 5 years after graduation (NA). Teeth were excluded from the study if pulp therapy was indicated as a result of total caries removal during CT (n = 2).

One day after the placement of the PMC (Kids Crown, Shinhung, Korea), the patients were contacted by a phone call to ascertain and record any acute complications. Afterwards, they were reviewed in our clinic every 6 months for 2 years.

#### Data collection

1. Baseline–The child’s age, gender, and social economic status (assigned according to school attended, type of insurance; income, parent education and job title) were recorded. The dmft was recorded by the operators.
2. Anxiety—The child’s anxiety was assessed and recorded using self-reported Facial Image Scale (FIS) [22]. Children had to select one from five faces, ranging from ‘very happy' score 1 to ‘very unhappy' score 5, which represented closely to how they felt at the time of assessment. The self-assessment was carried out before and at the end of each treatment. This assessment was repeated at the one-year review appointment.
3. Procedures time–It was recorded as the time when the operators started to carry out the clinical procedure until the operator felt they had finished.
4. Failure of PMCs–The PMC was assessed clinically at each review appointment, or at the time of emergency. Minor failures constituted dislodgement or perforation of the PMCs without pain. Major failures (clinical endpoint) constituted the teeth that caused pain and needed pulp therapy or extraction.
5. Periodontal health–It was assessed and recorded using the modified plaque index [23] and gingival index [24] around PMC margin.
6. Occlusal contact–It was assessed by passing an occlusal strip between the contra-lateral upper and lower posterior natural teeth when the child was asked to bite into maximum intercuspation. If the occlusal strip could pass without resistance between teeth anterior or posterior to the PMC, we considered the occlusion raised. If there was resistance to the teeth were considered to be in normal occlusion

#### Data analysis

The data were analysed using SPSS 20.0 for Windows (SPSS Inc, Chicago, Ill., USA). Descriptive statistics were used to report the frequency distributions of the evaluated variables. Comparison between groups were carried out using the Student t-test. Survival (primary endpoint), anxiety, plaque, gingival indices and occlusion were compared using Pearson Chi-Squared test (*χ*^2^) and Fisher’s exact test. In addition, survival analysis was carried out using the Kaplan–Meier and the log-rank test. A tooth was censored if the patients dropped out, or the tooth had exfoliated at the time of assessment. The censored date was the date when the patient was last seen. Only major failures of PMCs were considered for the purpose of this study. The null hypothesis was that there was no statistical difference (p> 0.05) in the survival rates of the treated teeth between HT and CT groups.

Incremental cost-effectiveness ratio (ICER) was calculated by dividing the difference in total costs incurred from each trial arm by the difference in the measure of health effect improvement from each arm. This provides a ratio of extra cost per extra unit of health effect [25,26] if any, and enables cost-effectiveness to be compared.

## Results

### Recruitment

**Fig 2:**
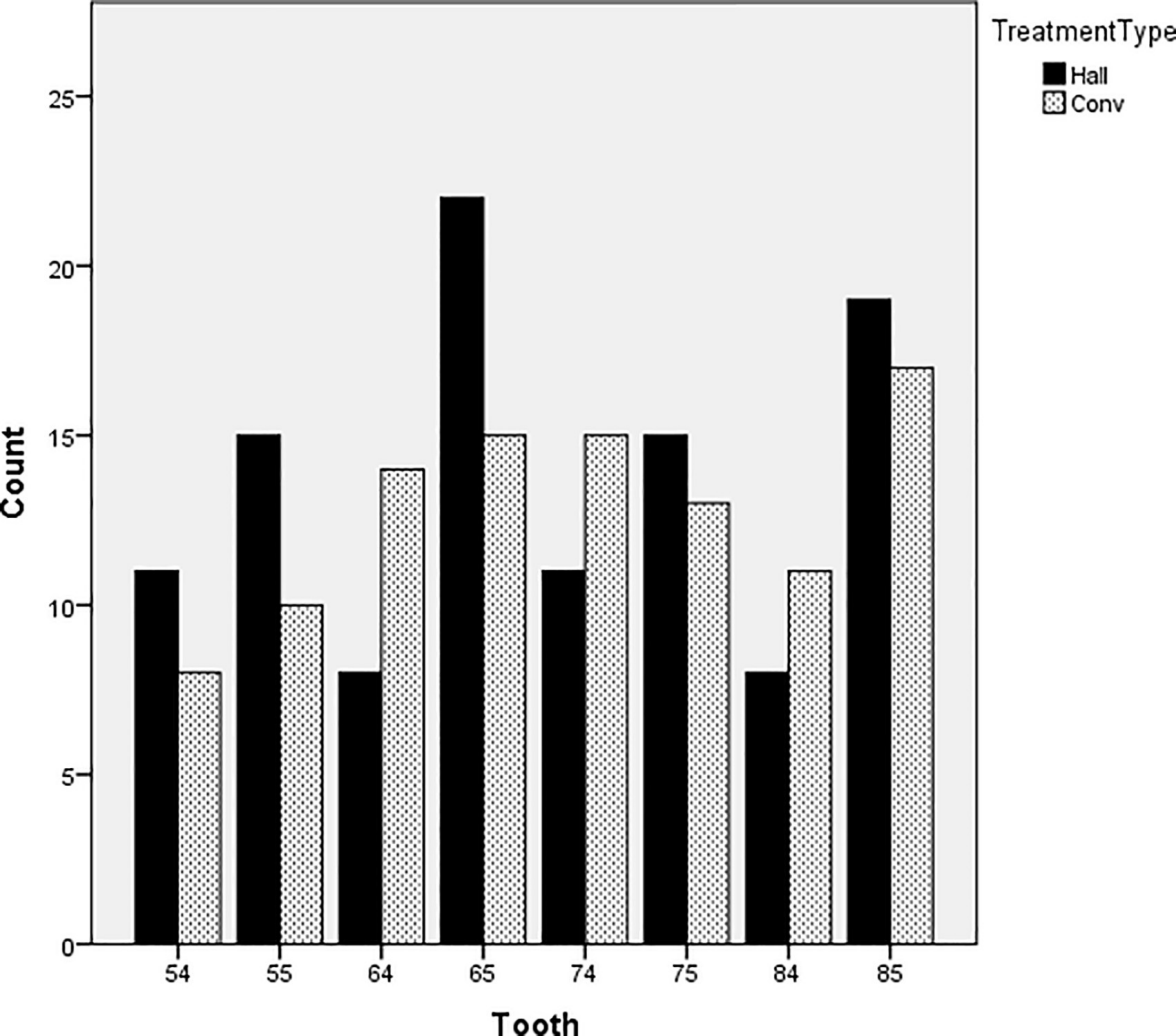
Distribution of the types of teeth treated in both groups (FDI notation).

After screening, patients who fulfilled the inclusion criteria were randomized until the minimum number of 73 teeth for each group was reached. One hundred and sixty-four patients were eventually recruited of which the HT arm had 86 children with 109 PMCs, and the CT group had 78 children with 103 PMCs. The number of PMCs that were lost to follow-up were 25 and 22 in the HT and CT groups, respectively. Clinical endpoints (i.e. major failures) were reached for 7 teeth from HT group and 6 teeth from CT group and follow-up was discontinued. Eventually, 84 and 81 PMCs in the HT and CT groups respectively were included in the analysis (Fig 1). The distribution of the type of teeth treated is shown in Fig 2. No obvious trend was observed between the 2 groups.

### Baseline

No statistically significant differences were found between the two caries treatment groups for age, gender, socioeconomic status, survival rates and caries experience (Table 1).

**Table 1:** Descriptive parameters describing each arm of the randomized clinical trial.

		HT (N = 109)	CT (N = 103)	p value
Age, mean (yrs) (SD; min-max)		6.89 (0.874; 5.00–8.27)	6.58 (0.850; 4.99–8.08)	NS^a^
Sex N (%)	Male	54 (49.5%)	53 (51.5%)	NS^a^
	Female	55 (50.5%)	50 (48.5%)	NS^a^
SES	Medium	19 (17.4%)	16 (15.5%)	NS^a^
	Low	90 (82.6%)	87 (84.5%)	NS^a^
Caries	dmft	6.08 (3.06)	6.33 (2.02)	NS^a^
	Occlusal	11 (10.0%)	7 (7.0%)	NS^a^
	Approximal	98 (90.0%)	96 (93.0%)	NS^a^
Mean procedure time in minutes (SD)		9.10 (2.87; 5.0–25.0)	33.91 (10.61; 23.0–85.0)	<0.001^a^
Survival rate at 24 months (%)		102 (93.6%)	97 (94.1%)	NS^b^
Minor failures		3 (2.7%)	6 (5.8%)	<0.05^b^
Major failures		7 (6.4%)	6 (5.8%)	NS^b^
Pulp exposure during carious tissue removal		0	2	-
Dropout rate (%)	6 months	4 (3.7%)	4 (3.9%)	NS^a^
	12 months	5 (4.6%)	8 (7.8%)	
	18 months	14 (12.8%)	13 (12.6%)	
	24 months	25 (22.9%)	22 (21.4%)	
Immediate postoperative complications (Lip biting, allergy, incorrect placement)		2 (1.8%)	4 (3.9%)	NS^a^
Tooth exfoliation		2 (1.8%)	1 (0.9%)	NS^a^

HT, Hall technique; CT, conventional technique; SES, socioeconomic status; dmft, decayed missing filled teeth; SD, standard deviation

^a^, student t-test

^b^, Fisher’s exact test; p>0.05 considered significant; NS, not significant.

### Anxiety

Table 2 shows the relation between anxiety categories, measured using FIS, and PMC placement methods. It was significantly different when assessed immediately postoperatively, *χ*^2^ (4, N = 212) = 21.04., p < 0.001, and after twelve months, *χ*^2^ (4, N = 212) = 52.74, p <0 .001. HT subjects were less likely to report anxiety than CT immediately or over time.

**Table 2:** Anxiety scores for the two groups using facial image scale.

	FIS	HT	CT	*χ*^2^ value	P value
Baseline	1	1	0	2.63	NS
	2	20	26		
	3	34	28		
	4	31	26		
	5	23	23		
Immediate post-op	1	1	1	21.94	<0.001
	2	43	44		
	3	64	39		
	4	1	8		
	5	0	11		
12 months post-op	1	24	0	52.94	<0.001
	2	18	12		
	3	26	7		
	4	21	47		
	5	15	33		

FIS, Facial image score ranging from 1 = very happy to 5 = very unhappy; HT, Hall technique; CT, conventional technique; p>0.05 considered significant; NS, not significant.

### PMC survival

**Fig 3:**
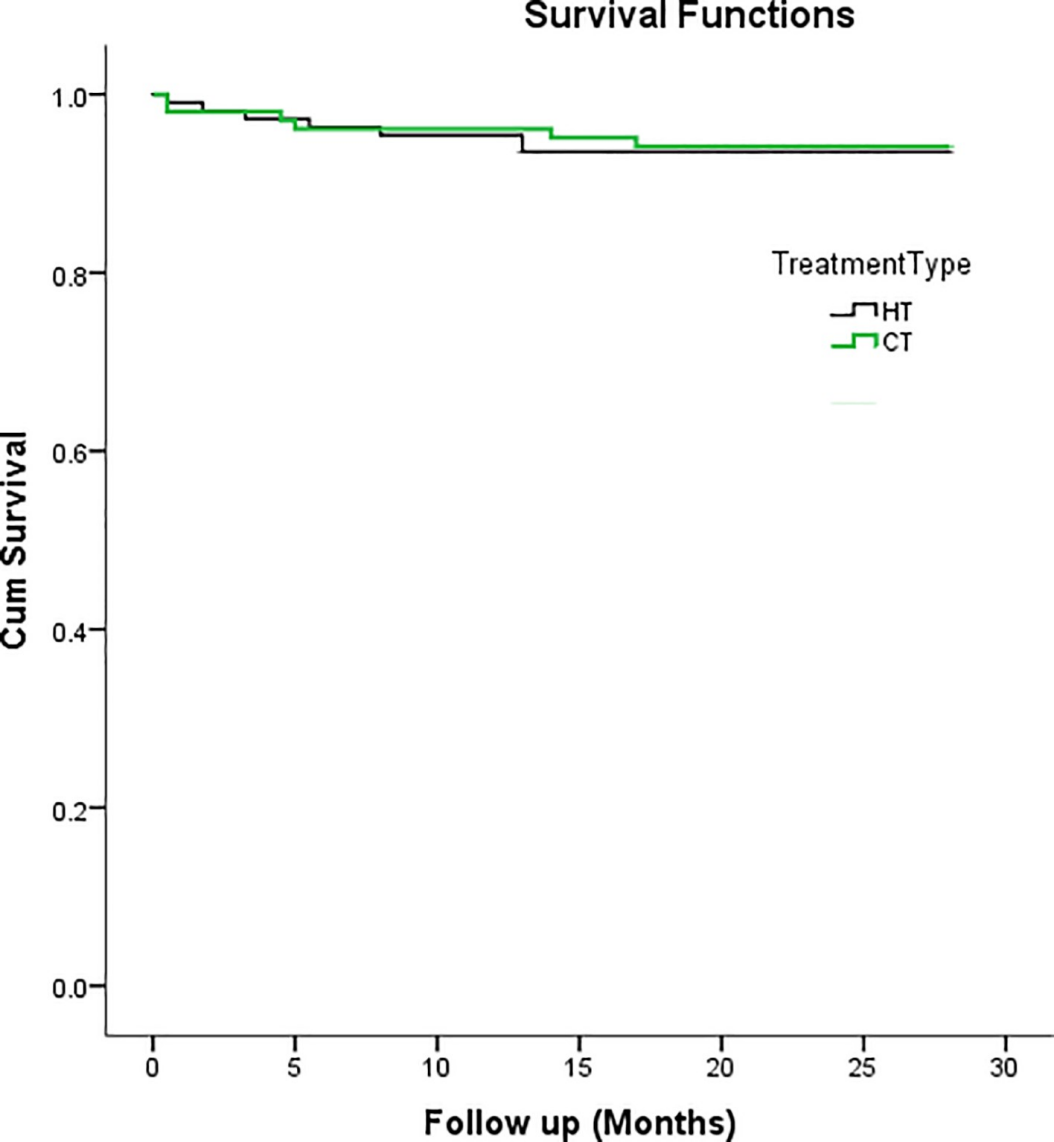
Kaplan-Meier survival function of PMCs for Hall technique (HT) and conventional technique (CT).

The drop-out rates at the end of the two years study was just over 20% for both groups with no significant differences between them. There was a significantly higher minor failure rate in the CT group (5.8%) than the HT group (2.7%), but they had similar major failure rates (5.8% and 6.4% respectively, Table 1). The survival rates for both groups were above 90% at 24 months (Fig 3). A chi-square test of independence was performed to examine the relation between PMC placement method and survival (primary endpoint) at 24 moths. The relation between these variables was not significant, *χ*^2^ (1) = 0.311, p >0.05. A log rank test was also run to determine if there were differences in the survival distribution for HT and CT. The survival distributions for the two interventions were not statistically significantly different, *χ*^2^ (1) = 0.007, p >0.05. The 1-year cumulative survival probabilities were 94.5% in the HT group and 96.0% in the CT group. The CT group had a slightly higher number of immediate post-operative complications (3.9%) compared to that in the HT group (1.8%) but the difference was not statistically significant due to small number. The complications include lip biting and pain. Of the teeth that had occlusal caries, no major failures were observed in CT group and one PMC was removed due to pain from HT group (Table 1). One patient was allergic to the PMC and was excluded from the study. Both groups had similar early exfoliation rate (Table 1).

### Periodontal health

Table 3 shows no significant relationship between PMC placement method (HT or CT) and periodontal health assessment represented by plaque index, *χ*^2^ (3) = 1.12, p > 0.05, and gingival index, *χ*^2^ (3) = 3.67, p > 0.05. Both PI and GI scores decreased over time in both groups.

**Table 3:** Assessment of periodontal health using the modified plaque index (Löe 1967), gingival index (Löe et al. 1972) and occlusion across time.

		HT					CT					Pearson Chi-squared test	
	Months	0 N = 109	6 N = 105	12 N = 104	18 N = 95	24 N = 84	0 N = 103	6 N = 99	12 N = 95	18 N = 90	24 N = 81	*χ*^2^ Value (df)	p value
Plaque Index	0	48 (44.0%)	66 (62.9%)	69 (66.3%)	71 (74.7%)	69 (82.1%)	55 (53.4%)	61 (61.6%)	66(69.5%)	64(71.1%)	61 (75.3%)	1.12 (3)	NS
	1	38 (34.9%)	33 (31.4%)	30 (28.8%)	21 (22.1%)	12 (14.3%)	32 (31.1%)	33 (33.3%)	25(26.3%)	23(25.6%)	17 (21.0%)		
	2	23 (21.1%)	6 (5.7%)	5 (4.8%)	3 (3.2%)	3 (3.6%)	16 (15.5%)	5 (5.1%)	4(4.2%)	3(3.3%)	3 (3.7%)		
Gingival index	0	62 (56.9%)	73 (69.5%)	74 (71.2%)	80 (84.2%)	78 (92.9%)	53 (51.4%)	68 (68.7%)	70(73.7%)	71(78.9%)	70 (86.4%)	3.67(3)	NS
	1	41 (37.6%)	26 (24.8%)	26 (25.0%)	12 (12.6%)	4 (4.8%)	39 (37.9%)	25 (25.3%)	20 (21.1%)	15 (16.7%)	8 (9.9%)		
	2	6 (5.5%)	6 (5.7%)	4 (3.8%)	3 (3.2%)	2 (2.4%)	11 (10.7%)	6 (6.1%)	5 (5.3%)	4 (4.4%)	3 (3.7%)		
Occlusion	Normal	0	102 (97.1%)	104 (100%)	95 (100%)	84(100%)	99 (96.1%)	99 (100%)	95 (100%)	90 (100%)	81 (100%)	188.77(2)	<0.001
	Raised	109 (100%)	3 (2.9%)	0	0	0	4 (3.9%)	0	0	0	0		

HT, Hall technique; CT, conventional technique; df, degrees of freedom, p>0.05 considered significant; NS, not significant.

### Occlusal contact

In the HT group, nearly all the children had raised occlusions immediately after placement of PMCs (Table 3). Only 4% of the children in CT group had raised occlusal contacts postoperatively. At 6 months, nearly all the children returned to normal occlusal contacts except 3% in HT group. By 12 months, all the children had normal occlusion.

### Cost effectiveness

The mean procedure time for the CT group (33.9 minutes; SD = 10.61) was significantly higher (p<0.001) than that in the HT group (9.1 minutes; SD = 2.87), Table 1. The calculated mean cost per unit for HT was US$2.45 (SD = 0.14), almost one-third cheaper than the cost of US$7.81 (SD = 0.14) for the CT. The ICER (incremental cost-effectiveness ratio) was US$136.56 more for each PMC placed by CT compared to that placed by HT per life year, Table 4.

**Table 4:** Cost comparison between the two modalities of treatment.

	HT	CT	p value
Total cost	$267.58	$804.47	
Total successful treatments	96	95	
Major failures	6	5	
Mean cost per unit (±SD)	$2.45 (±0.14)	$7.81 (±1.41)	<0.05
Cost per procedure (US$)	$2.79	$8.47	
ICER (US$/per year of survival)	-	$136.56	

ICER = incremental cost-effective ratio

## Discussion

### Setting

This is the first prospective RCT comparing the efficacy of the PMCs placed by Hall and conventional techniques in an under-developed country. Little has been reported on the use of preformed metal crowns from developing countries. Sudan is one of the poorest countries in the world with internal conflicts and political instability [27]. In a country of 40 million, very few dentists exist outside the capital. The United Nations and WHO estimate that over 40% of the population in Sudan to be under 15 years of age [28,29]. All Counties provide dental services through dental therapists. It is common for dental clinics to operate without amenities such as running water and electricity. The burden of dental caries is high. Tooth extractions are common as patients afford little else. In this setting, the limited resources have to be managed efficiently in terms of treatment efficacy, survival, and cost-effectiveness in relation to materials, human resources, education, supply chains and failure management [30].

The present study used two clinicians with different grades and experiences for each technique for the following reasons:

1. It enabled the comparison of the two techniques in terms of cost effectiveness and efficacy. Most of the previous studies on placement of preformed metal crowns were carried out by dentists, and mainly with the conventional technique. Hence, it is usually regarded as the “gold standard”. This is the first study in which a dental therapist was used to place the crowns with HT so that potential reduced cost could be investigated if the efficacies for both techniques are similar, as shown in the results.
2. This design also reduced the potential unconscious bias of favouring a particular technique. As mentioned before, placing PMC using the CT is generally regarded as the “gold standard” and it is difficult to change the preconception of an experienced dentist who had been using CT for many years. As the technique cannot be blinded, unconscious bias might be introduced as the dentist might modify, or be reluctant to use the HT, in the belief that CT gives the better outcome.

### Survival of PMCs

The present study confirms the high success rate of PMCs, with more than 90% cumulative probability of surviving over 24 months. It is comparable to the survival rate (95%) of PMCs placed by HT in developed countries [6,31]. The PMCs are shown to have longer survival rates than other restorative materials in primary molars [6,17,32–36]. The non-significant difference between the HT and CT agrees with the results from other retrospectives studies [13,14]. When major and minor failures were combined, the success was 90.8% and 88.3% for HT and CT respectively, in line with other investigators who show similar success rates of about 87% [37]. Although one *in vitro* study [38] reported that HT PMCs have more micro-leakage compared to the CT PMCs, the present clinical study does not show that as it has not affected the survival rate of the PMC.

### Periodontal health and occlusion

It has been a concern that a PMC, if it is placed using the HT, could cause deterioration of periodontal health [39], and/or cause occlusal discrepancies. However, the present study shows that plaque and gingival indices improved over time for both groups. The high level of poor oral hygiene and gingival inflammation at baseline might be due to the children’s/carers’ initial ignorance of oral health. We are unable to establish whether the improvements were due to raised awareness and/or motivation, however, the PMCs might enable the children to maintain better plaque control [40]. Although periodontal depths were not measured, the improved PI and GI after 12 months, and nearly all the raised occlusion returned to normal after 6 months, indicate that the PMC did not cause any periodontal damages, even with the HT. It has been shown that the raised occlusion could be re-established after 1 month [41]. Nevertheless, further studies may be needed to assess the long-term effects of PMCs on vertical dimension, facial height and malocclusion.

### Anxiety

The present study agrees with previous studies that the children’s anxieties are reduced immediately after treatment [42,43]. However, the HT group had lower postoperative anxiety scores than the CT group. This is different to the children’s acceptance of treatment reported by BaniHani et al. [13] who show no differences between the two techniques. We also show that the HT had significantly lower anxiety level than baseline, whereas the children seemed to remember the unpleasantness associated with the CT placement of PMCs and had a higher anxiety score after 12 months. This is in line with previous reports that show negative memories of painful first procedures are linked to greater distress in future procedures and children tend to develop exaggerated anxiety and distress due to the pain memory [44,45]. The use of local anaesthetic and the noisy air turbine operated high-speed handpieces might also be the cause of the increased anxiety in the CT group.

### Cost effectiveness

The present RCT demonstrates that the PMCs placed by the HT are more cost-effective than CT (Table 4). It has been estimated that conventionally fitted PMCs cost 35% less than amalgam restorations [3]. A study from Germany also confirms that HT PMCs are more cost-effective than conventional restorations [46]. In the UK, total cumulative costs were significantly lower in HT than conventional restorations (24 and 29 GBP, respectively) [47]. In our setting in Sudan, PMCs placed by CT were three times more expensive than by HT. Apart from the significantly reduced time of procedures by HT, the PMCs were placed by a dental therapist, whose salary was much lower than that of a dentist. However, the effectiveness of treatment was similar, resulting in an ICER of $136.52 for CT intervention compared to HT per year of survival. It is also noted that PMCs can be placed using HT during frequent shortages of electricity and water supply, and shortages even without the need for a dental chair.

### Clinical implications

Despite the high success rate and effectiveness of PMC in children with high caries risk, dentists from general dental practice do not routinely use PMCs for managing carious primary molars [8,12,48]. Reasons for not using the technique include lack of training, underfunding, perceived lack of cost-effectiveness in general practice, and patient cooperation [49,50]. In the present study, a dental therapist, with 2 years clinical experience, is able to place the PMCs using the HT with minimal training, and had similar clinical performance to the CT groups. The HT was also shown to be more advantageous in term of time, cost, and anxiety of the children. Hence, more therapists should be trained to place HT PMCs in general practices, and in rural communities [51], in order to improve health outcomes and tooth survival. One further advantage of the HT is that not only PMCs can be placed without electricity, but also in the “field”, e.g. in schools, without a dental chair. This might be important in particular in under-developed countries.

### Limitations

In this randomized clinical trial, the failures were assessed clinically in terms of pain and sepsis. No bitewing radiographs were taken to assess success/failure and no teeth with pulpal involvement (i.e. no pain/sepsis or radiographic evidence) were included. Hence, the survival rates in this study may have been over-estimated due to the lack of postoperative radiographs assessing pulp status. However, as the patients were randomized, the comparison between the two techniques should still be valid.

The reported cost-effectiveness for HT did not consider the cost required for diagnosis by a dentist. In Sudan, therapists can diagnose and treat carious lesions without pulp involvement. We appreciate that different health systems exist and the remit of dental therapist from this study may not apply in other settings. Furthermore, some children required specialist treatment for some failures. The study did not consider the impact of managing post-operative major or minor failures of PMCs on ICER. This includes extractions, pulp therapy and future orthodontic intervention.

About one-fifth of the sample dropped out from the study. This is unlikely to have an impact as there was sufficient statistical power to test the differences. The drop-out rate is probably associated with economic hardship rather than treatment failure. This is because our clinic is the only facility that provides free PMCs as part of the routine care for children with low SES. Those who seek treatment elsewhere will incur significant cost. The impact of aesthetics on children and parents were not examined. However, a survey shows that 84% of parent respondents had no concerns about PMC's colour, and 56% of children liked the colour of the PMC [52].

## Conclusion

PMCs placed using the Hall or conventional techniques have excellent survival in deprived communities. HT is highly cost-effective in terms of materials, labour and time. HT induces less self-reported anxiety than the more invasive CT. Placing PMC using HT by therapists is a successful and cost-effective public health intervention for carious primary molars in communities and developing countries.

## Supporting information

S1 File CONSORT 2010 checklist. (DOC)

S2 File SSC.Sudan trial protocol. (DOCX)

## References

[1] Chisii LA, Collares K, Cademartori MG, de Oliveira LJC, Conde MCM, Demarco FF, et al Restorations in primary teeth: a systematic review on survival and reasons for failures. International Journal of Paediatric Dentistry. 2018; 28: 123–139. 10.1111/ipd.12346 29322626

[2] Dawson LR, Simon JF, Taylor PP. Use of amalgam and stainless steel restorations for primary molars. ASDC J Dent Child.2018; 48: 420–2. Available: http://www.ncbi.nlm.nih.gov/pubmed/6946083 6946083

[3] Eriksson AL, Paunio P, Isotupa K. Restoration of deciduous molars with ion-crowns: retention and subsequent treatment. Proc Finn Dent Soc. 1988;84: 95–9. Available: http://www.ncbi.nlm.nih.gov/pubmed/3134654 3134654

[4] Wong FS, Day SJ. An investigation of factors influencing the longevity of restorations in primary molars. J Int Assoc Dent Child. 1990;20: 11–6. Available: http://www.ncbi.nlm.nih.gov/pubmed/2074359 2074359

[5] Curzon MEJ, Roberts JF. Kennedy’s paediatric operative dentistry. Wright; 1996.

[6] Innes NPT, Evans DJP, Stirrups DR. Sealing caries in primary molars: Randomized control trial, 5-year results. J Dent Res. 2011;90: 1405–1410. 10.1177/0022034511422064 21921249

[7] Roberts A, McKay A, Albadri S. The use of Hall technique preformed metal crowns by specialist paediatric dentists in the UK. Br Dent J. 2018; 28: 123–139. 10.1038/sj.bdj.2018.4 PMC5770477 29326453

[8] Threlfall AG, Pilkington L, Milsom KM, Blinkhorn AS, Tickle M. General dental practitioners’ views on the use of stainless steel crowns to restore primary molars. Br Dent J. 2005; 199: 453–455. 10.1038/sj.bdj.4812746 16215580

[9] Schüler IM, Hiller M, Roloff T, Kühnisch J, Heinrich-Weltzien R. Clinical success of stainless steel crowns placed under general anaesthesia in primary molars: An observational follow up study. J Dent. 2014; 42: 1396–1403. 10.1016/j.jdent.2014.06.009 24994618

[10] Halawany HS, Salama F, Jacob V, Abraham NB, Moharib TN Bin, Alazmah AS, et al A survey of pediatric dentists’ caries-related treatment decisions and restorative modalities–A web-based survey. Saudi Dent J; 2017; 29: 66–73. 10.1016/j.sdentj.2017.03.001 28490845 PMC5411897

[11] Kindelan SA, Day P, Nichol R, Willmott N, Fayle SA. UK National Clinical Guidelines in Paediatric Dentistry: stainless steel preformed crowns for primary molars. Int J Paediatr Dent. 2008; 18 Suppl 1: 20–28. 10.1111/j.1365-263X.2008.00935.x 18808544

[12] Innes NPT, Ricketts D, Chong LY, Keightley AJ, Lamont T, Santamaria RM. Preformed crowns for decayed primary molar teeth. Cochrane Database Syst Rev. 2015:Issue 12, Art. No. 005512. 10.1002/14651858.CD005512.pub3 26718872 PMC7387869

[13] BaniHani A, Duggal M, Toumba J, Deery C. Outcomes of the conventional and biological treatment approaches for the management of caries in the primary dentition. Int J Paediatr Dent. 2018; 28: 12–22. 10.1111/ipd.12314 28691235

[14] Ludwig KH, Fontana M, Vinson LQA, Platt JA, Dean JA. The success of stainless steel crowns placed with the Hall technique :A retrospective study. J Am Dent Assoc. American Dental Association; 2014;145: 1248–1253. 10.14219/jada.2014.89 25429038

[15] Enderlein G. Pocock S. J.: Clinical Trials—a practical approach. John Wiley & Sons, Chichester—New York—Brisbane—Toronto—Singapore 1983, 265 S., £ 16.95. Biometrical J. 1985;27: 634–634. 10.1002/bimj.4710270604

[16] Kim J, Seo BS. How to calculate sample size and why. Clin Orthop Surg. 2013;5: 235–242. 10.4055/cios.2013.5.3.235 24009911 PMC3758995

[17] Innes NPT, Stirrups DR, Evans DJP, Hall N, Leggate M. A novel technique using preformed metal crowns for managing carious primary molars in general practice—a retrospective analysis. Br Dent J. Nature Publishing Group; 2006;200: 451 10.1038/sj.bdj.4813466 16703041

[18] Santamaria RM, Innes NPT, Machiulskiene V, Evans DJP, Splieth CH. Caries management strategies for primary molars: 1-yr randomized control trial results. J Dent Res. 2014;93: 1062–9. 10.1177/0022034514550717 25216660 PMC4293767

[19] Milsom KM, Kearney-Mitchell PI, Shahid S, Threlfall A, Blinkhorn A, Tickle M. The treatment of primary molar teeth presenting with two surface caries—A review of care delivered by the Personal Dental Services in Bradford. Int J Heal Promot Educ. Taylor & Francis Group; 2006;44: 141–144. 10.1080/14635240.2006.10708087

[20] Papathanasiou a G, Curzon ME, Fairpo CG. The influence of restorative material on the survival rate of restorations in primary molars. Pediatr Dent. 1990;16: 282–8. 7937261

[21] Rennie D. CONSORT revised—improving the reporting of randomized trials. JAMA. 2001;285: 2006–7. Available: http://www.ncbi.nlm.nih.gov/pubmed/11308440 11308440 10.1001/jama.285.15.2006

[22] Buchanan H, Niven N. Validation of a Facial Image Scale to assess child dental anxiety. Int J Paediatr Dent. 2002;12: 47–52. 10.1046/j.0960-7439.2001.00317.x 11853248

[23] Löe H. The gingival index, the plaque index and the retention index systems. J Periodontol. Am Acad Periodontology; 1967;38: 610–616. 10.1902/jop.1967.38.6.610 5237684

[24] Löe H, Fehr FR, Schiött CR. Inhibition of experimental caries by plaque prevention. Eur J Oral Sci. Wiley Online Library; 1972;80: 1–9. 10.1111/j.1600-0722.1972.tb00257.x 4502580

[25] Detsky AS, Naglie IG. A clinician’s guide to cost-effectiveness analysis. Ann Intern Med. 1990;113: 147–154. 10.7326/0003-4819-113-2-147 2113784

[26] Hesse D, de Araujo MP, Olegário IC, Innes N, Raggio DP, Bonifácio CC. Atraumatic Restorative Treatment compared to the Hall Technique for occluso-proximal cavities in primary molars: study protocol for a randomized controlled trial. Trials. 2016;17: 169 10.1186/s13063-016-1270-z 27029801 PMC4815168

[27] A Poverty Profile for the Northern States of Sudan The World Bank Poverty Reduction and Economic Management Unit, Africa Region. 2011.

[28] WHO | Sudan. WHO. World Health Organization; 2018; Available: http://www.who.int/countries/sdn/en/

[29] World Population Prospects The 2017 Revision [Internet]. Available: https://esa.un.org/unpd/wpp/Publications/Files/WPP2017_KeyFindings.pdf

[30] Petersen PE, Bourgeois D, Ogawa H, Estupinan-Day S, Ndiaye C. The global burden of oral diseases and risks to oral health. Bulletin of the World Health Organization. 2005; 83: 661–669. doi: /S0042-96862005000900011 16211157 PMC2626328

[31] Innes NP, Evans DJP, Stirrups DR. The Hall Technique; A randomized controlled clinical trial of a novel method of managing carious primary molars in general dental practice: Acceptability of the technique and outcomes at 23 months. BMC Oral Health. 2007; 20: 7:18. 10.1186/1472-6831-7-18 PMC2265270 18096042

[32] Ram D, Fuks AB, Eidelman E. Long-term clinical performance of esthetic primary molar crowns. Pediatr Dent. 2003; 25: 582–4. Available: http://www.ncbi.nlm.nih.gov/pubmed/14733474 14733474

[33] Atieh M. Stainless steel crown versus modified open-sandwich restorations for primary molars: A 2-year randomized clinical trial. Int J Paediatr Dent. 2008;18: 325–332. 10.1111/j.1365-263X.2007.00900.x 18328050

[34] Santamaria RM, Innes NPT, Machiulskiene V, Evans DJP, Splieth CH. Caries management strategies for primary molars: 1-yr randomized control trial results. J Dent Res. 2014;93: 1062–9. 10.1177/0022034514550717 25216660 PMC4293767

[35] Hutcheson C, Seale NS, McWhorter A, Kerins C, Wright J. Multi-surface composite vs stainless steel crown restorations after mineral trioxide aggregate pulpotomy: a randomized controlled trial. Pediatr Dent. 2012; 34: 460–7. Available: http://www.ncbi.nlm.nih.gov/pubmed/23265162 23265162

[36] Santamaría RM, Innes NPT, Machiulskiene V, Schmoeckel J, Alkilzy M, Splieth CH. Alternative Caries Management Options for Primary Molars: 2.5-Year Outcomes of a Randomised Clinical Trial. Caries Res. 2017;51: 605–614. 10.1159/000477855 29258064

[37] Tseveenjav B, Furuholm J, Mulic A, Valen H, Maisala T, Turunen S, et al Survival of extensive restorations in primary molars: 15-year practice-based study. Int J Paediatr Dent. 2018;28: 249–256. 10.1111/ipd.12348 29205613

[38] Erdemci ZY, Cehreli SB, Tirali RE. Hall versus conventional stainless steel crown techniques: in vitro investigation of marginal fit and microleakage using three different luting agents. Pediatr Dent. 36: 286–90. Available: http://www.ncbi.nlm.nih.gov/pubmed/25197992 25197992

[39] Belduz Kara N, Yilmaz Y. Assessment of oral hygiene and periodontal health around posterior primary molars after their restoration with various crown types. Int J Paediatr Dent. 2014; 24: 303–313. 10.1111/ipd.12074 24164167

[40] Jeffcoat M. Chemical plaque control: how do you advise your patients? Int Dent J. 1993;43: 415–21. Available: http://www.ncbi.nlm.nih.gov/pubmed/8282424 8282424

[41] van der Zee V, van Amerongen WE. Short communication: Influence of preformed metal crowns (Hall technique) on the occlusal vertical dimension in the primary dentition. Eur Arch Paediatr Dent. 2010;11: 225–7. Available: http://www.ncbi.nlm.nih.gov/pubmed/20932395 20932395 10.1007/BF03262751

[42] Holmes RD, Girdler NM. A study to assess the validity of clinical judgement in determining paediatric dental anxiety and related outcomes of management. Int J Paediatr Dent. 2005;15: 169–76. 10.1111/j.1365-263X.2005.00633.x 15854112

[43] Mahiepala NA, Phan VL, Kieu KD, Koppen JPL, Hussain BH, Huang B. Influencing factors of paediatric dental anxiety levels in an undergraduate dental clinic. Eur J Paediatr Dent. 2015;16: 159–62. Available: http://www.ncbi.nlm.nih.gov/pubmed/26147825 26147825

[44] Chen E, Zeltzer LK, Craske MG, Katz ER. Alteration of memory in the reduction of children’s distress during repeated aversive medical procedures. J Consult Clin Psychol. 1999;67: 481–90. Available: http://www.ncbi.nlm.nih.gov/pubmed/10450618 10450618 10.1037//0022-006x.67.4.481

[45] Rocha EM, Marche TA, von Baeyer CL. Anxiety influences children’s memory for procedural pain. Pain Res Manag. 2009; 14: 233–7. Available: http://www.ncbi.nlm.nih.gov/pubmed/19547763 19547763 10.1155/2009/535941 PMC2706554

[46] Schwendicke F, Stolpe M, Innes N. Conventional treatment, Hall Technique or immediate pulpotomy for carious primary molars: a cost-effectiveness analysis. Int Endod J. 2016; 49: 817–826. 10.1111/iej.12537 26331379

[47] Schwendicke F, Krois J, Robertson M, Splieth C, Santamaria R, Innes N. Cost-effectiveness of the Hall Technique in a Randomized Trial. J Dent Res. 2019;98: 61–67. 10.1177/0022034518799742 30216734

[48] Tickle M, Threlfall AG, Pilkington L, Milsom KM, Duggal MS, Blinkhorn AS. Approaches taken to the treatment of young children with carious primary teeth: a national cross-sectional survey of general dental practitioners and paediatric specialists in England. Br Dent J. 2007;203: E4; discussion 102–3. 10.1038/bdj.2007.570 17571091

[49] Maggs-Rapport FL, Treasure ET, Chadwick BL. Community dental ocers’ use and knowledge of restorative techniques for primary molars: an audit of two Trusts in Wales. Int J Paediatr Dent. 2000;10: 133–139. 11310098 10.1046/j.1365-263x.2000.00182.x

[50] Chadwick BL, Gash C, Stewart K. Preformed metal crowns: views of a group of dental practitioners in North Wales. Prim Dent Care. 2007;14: 140–4. 10.1308/135576107782144351 17931495

[51] Nash DA, Friedman JW, Mathu-Muju KR, Robinson PG, Satur J, Moffat S, et al A review of the global literature on dental therapists. Community Dent Oral Epidemiol. 2014;42: 1–10. 10.1111/cdoe.12052 23646862

[52] Roberts JF, Attari N, Sherriff M. The survival of resin modified glass ionomer and stainless steel crown restorations in primary molars, placed in a specialist paediatric dental practice. Br Dent J J. 2005;198: 427–431. 10.1038/sj.bdj.4812197 15870802

