# Supplementary material for: A randomized clinical trial comparing Hall vs conventional technique in placing preformed metal crowns from Sudan
Source: PLoS One. 2019 Jun 3;14(6):e0217740. doi: 10.1371/journal.pone.0217740 (PMC6546341; doi:10.1371/journal.pone.0217740)

Protocol Cover Page

**Protocol Title:** A randomized clinical trial comparing Hall and conventional preformed metal crown placement techniques from Sudan.

**Protocol Number:** Paed/Dent/11-01

**Protocol Date:**  18/11/2014

**Study Phase:** I

**Project Leader:** F Elamin PhD

**Protocol Author(s):** F Elamin PhD

F Wong PhD

#### **INVESTIGATOR:**

Dr F Elamin

Khartoum Centre for Research and Medical Training

Islamic Bank Building

Khartoum

Sudan

Email: fadilelamin@yahoo.co.uk

#### **SPONSOR**:

Khartoum Centre for Research and Medical Training

Islamic Bank Building

Khartoum

Sudan

# Confidentiality Agreement

This document is a confidential communication of KCRMT. Acceptance of this document constitutes the agreement by the recipient that no unpublished information contained within will be published or disclosed without prior written approval, except that this document may be disclosed to the appropriate Ethics Committee and Regulatory Authority under the condition that they are requested to keep it confidential.

# TABLE OF CONTENTS PAGE

LIST OF ABBREVIATIONS AND DEFINITIONS 4

STUDY CONTACT LIST 5

PROTOCOL SYNOPSIS 6

1 INTRODUCTION 8

- 1. Background 8
  2. Study Rationale 8

1. STUDY OBJECTIVES 8
2. STUDY PLAN AND PROCEDURES 8
   1. Study design 8
      1. Scheduled clinic visits 9
      2. Visits and assessments 9
   2. Study population 9
      1. Inclusion criteria 9
      2. Exclusion criteria 10
      3. Justification for inclusion and exclusion criteria 10
      4. Criteria for discontinuation 10
   3. Investigational Products and Treatments 10
      1. Treatment Schedule 10
      2. Randomization 10
      3. Identity of Study Products 10
      4. Storage and Accountability 10
      5. Allowed restorative treatment and medication 11
      6. Compliance 11
3. STUDY MEASUREMENTS AND ENDPOINTS 11
   1. Primary Safety Endpoints 11
   2. Secondary Safety Endpoint 11
   3. Measurements at each visit 11
   4. Specific detail on measurements 11
      1. Adverse Events
      2. Clinical Examination 12
      3. Radiographic Examination 12

1. STATISTICAL METHODS 13
   1. Determination of sample size 13
   2. Statistical analysis 13
   3. Changes to the protocol 13
2. ETHICS 13
   1. Ethics review 13
   2. Ethical conduct of the study 13
   3. Subject information and consent 13
   4. Subject data protection 14
3. DATA QUALITY ASSURANCE 14
4. STUDY TIMETABLE AND TERMINATION 14
5. REFERENCES 15
6. APPENDICES
   1. Declaration of Helsinki 16
   2. Sample written information and informed consent (with Arabic translation) 20
   3. Clinical Outcome Criteria 27

# LIST OF ABBREVIATIONS AND DEFINITIONS

The following abbreviations and specialist terms are used in this study protocol:

### Abbreviation Explanation

AE Adverse Event

CT Conventional technique

CRF Case Report Form

GCP Good Clinical Practice

HT Hall technique

ICER Incremental cost effectiveness reatio

ICH International Conference of Harmonisation

IEC Independent Ethics Committee

IRB Independent Review Board

KCRMT Khartoum Centre for Research and Medical Training

N/A Not applicable

PMC Preformed metal crown

### Definitions

*Hall technique:*

*Conventional Technique:*

## STUDY CONTACT LIST

**For questions regarding the conduct of this study, please contact:**

**Project Leader:** Dr Fadil Elamin PhD

E-mail: fadilelamin@yahoo.co.uk

Mobile: 00249123303311

**Clinical Trial Manager:** Dr Nihal Abdelazim MSc

**(Clinical investigator)** E-mail: nihalabdelazeem@gmail.com Telephone: 00249918264131

**For reporting a serious adverse event, please contact:**

**Clinical Trial Manager:** Dr Fadil Elamin

E-mail: fadilelamin@yahoo.co.uk Telephone: 00249123303311

**For questions regarding data quality assurance, please contact:**

# Independent Study Monitor: Ms Mayada Emam

Email. khartoumcentre@gmail.com

Tel. 00249922805635

### PROTOCOL SYNOPSIS

| **TITLE** | A randomized clinical trial comparing Hall and conventional preformed metal crown placement techniques from Sudan. | | |
| --- | --- | --- | --- |
| **Investigational site:** | Dental Department  Islamic Bank Building  Khartoum  Sudan | | |
| **Investigators:** | Dr Fadil Elamin, Dr Nihal Abdelazim, Dr Youra Mirghani, Prof FSL Wong | | |
| **Sponsor:** | Khartoum Centre for Research and Medical Training (KCRMT) | | |
| **Representatives:** | Dr Fadil Elamin | | |
| **Study number:** | Paed/Dent/11-01 | | |
| **Final Protocol:** | Nov 2014 |  |  |
| **Ethics Approval:** | Jan 2015 | **Statistical analysis:** | **April 2018** |
| **Clinical Phase:** | Feb 2015 to March 2016 | **Study Report:** | May 2018 |
| **OBJECTIVES:** | To investigate the viability of restoring carious primary teeth using the biological Hall technique in relation to convential methods of placing preformed metal crowns. | | |
| **STUDY DESIGN:** | A randomized controlled study over a 24-month period. Volunteers will be screened at Visit 1 and eligible subjects will be randomized to either *the Hall Technique group or the Conventional Treamenent group.* Visits 2, 3, 4 and 5 (final) will be scheduled at 6 monthly intervals. Dentists will enter clinical outcomes and events. Additionally, the first visit will include pre- and postoperative anxiety assessements using facial image scale (REF). Anxiety scores will be repeated six monthly. | | |
| **SUBJECTS:** | Inclusion criteria  - Medically fit - Compliance: ablity to undergo examaination unassisted - Tooth previously unrestored - No pain - Class I or Class II carious cavity in one or two primary molars teeth - Absence of clinical a history and signs of pulp necrosis and infection - Radiographic evidence caries prior to restorative treatment  Exclusion criteria  - Demographic inaccessabilty - Poor co-operation and inability to undergo clinical examination unaided - Historic or ongoing pain - Evidence of clinical pulp involvement: active sinus, abscess - Evidence of radiographic pulp involvment | | |
| **PRODUCT TO BE EVALUATED** | **Test:** *PMCs fitted using HT*  **Reference group:** PMCs fitted using CT | | |
| **DURATION OF STUDY** | 24 months | | |
| **ENDPOINTS:** | **SAFETY**   1. Adverse events 2. Failure to attend 3. Dental abscess 4. Allergies 5. Severe pain 6. Psychological trauma | | |
| **SAMPLE** **SIZE:** | 158 subjects | | |
| **STATISTICAL ANALYSIS**: | Summary statistics will describe group differences using student T-test.  Survival curves will be used to demonstrate and compare survival between the two treatment modalities. Cost will be compared between the and ICER calculated. | | |

# 1. INTRODUCTION

**Background**

Preformed metal crowns are effective techniques for the management of caries in children with longterm preservation of the primary molar and its function. Biological methods (1, 2) such as the Hall technique have potential of changing how caries is managed in underdevolped countries and communitiesPreformed metal crwon placement is viewed as a specialist technique that is genrally not justified in being placed in poor settings. Conventional preparation and placement continues to be taught in dental schools but is not expected to be taken up following graduation in much of the developing world. Ease of placing PMCs using biological methods such as the Hall technique may offer an effective management tool for caries management in poor communities. Unfortunately no controlled trials exist to show if using biolgogical techniques for caries management combined with PMC placement using Hall techniques are detrimental to primary tooth survival.

**1.2 Study Rationale**

There has been no properly conducted prospective clinical trials to compare Hall technique with other forms of PMC placement. Few trials exist that show that the technique is superior to other forms of caries management. All these studies are from developing countries where facilities and expertise are available. Despite the high burden of dental caries in children from developing countries managing caries risk usually has low priority. As there is currently there is no evidence for against cost-effectiveness, health authorities can not be expected to implement public health measures that have not been thouroughly proven to work and side effects taken into account.

Given the widespread prevalence of caries in developing countries effective strategies that address public health needs should be investigated. In addition to survival cost effectiveness is the issue most likely to determine uptake in developing countries.

**2. STUDY OBJECTIVES**

The primary objective of this study is to investigate the decayed primary tooth survival using the biological Hall technique as a viable treatment options in deprived settings.

A secondary objective is to investigate the sensitivity of the technique to being carried out by auxillary allied dental clinicians such as therapists. Dental treatment induces considerable anxiety and is an important parameter for children and clinicians during routine care. Stress induced by carrying out dental procedures in children will be assessed using self assessed facial image scale questionnairres.

##### 3. STUDY PLAN AND PROCEDURES

##### 3.1 Study design

This is a 24 months long, randomized, blind controlled study to evaluate survival and effectiveness of the Hall technique comared to Cenventional techniques for fitting PMCs. Subjects must be medically healthy children between 5-8 years of age with more than one carious primary molar. Consent should be sought and obtained from accompanying parent. The inclusion and exclusion criteria are listed below. Complying subjects will be randomized to receive a preformed metal crown using either the Hall technique or by the Conventional technique, as defined above. Self perception question

The study will be conducted at the Dental Clinic, Islamic Bank Building, Qasr Street, Khartoum, Sudan.

**3.1.1 Scheduled clinic visits**

| **Visit number** | **Type of visit** | **Month number (& Months between visits)** |
| --- | --- | --- |
| Visit 1 | Information, Screening & Consent; Randomization;  Pre and post perceived anxiety level questionnaire (FIS) (3); clinical treatment; Measure clinical outcomes; Measure treatment time. |  |
| Visit 2 | Measure clinical outcomes and anxiety | 6 months |
| Visit 3 | Repeat measures of clinical outcomes and anxiety | 12 months |
| Visit 4 | Repeat measures of clinical outcomes and anxiety | 18 months |
| Visit 5 | Repeat measures of clinical outcomes and anxiety | 24 months |

### Visits and assessments

| **Week** | **0** | **6** | **12** | **18** | **24** |
| --- | --- | --- | --- | --- | --- |
| **Visit** | **1** | **2** | **3** | **4** | **5** |
| Informed consent | X |  |  |  |  |
| Procedure time | X |  |  |  |  |
| Pre-operative facial image scale | X |  |  |  |  |
| Post-operative facial image scale | X | X | X | X | X |
| Occlusion | X | X | X | X | X |
| Plaque index | X | X | X | X | X |
| Gingival index | X | X | X | X | X |

**3.2 Study population**

The study population will be healthy child volunteers who will be mainly recruited from children (5-8 years) attending the dental clinic for routine care with dental insurance to cover the prescribed procedure. The investigator should complete a subject screening log to document subjects considered for enrolment, but never enrolled to establish that the subject population is selected without bias. Recruitment will continue until 158 subjects are randomized into the 2 groups of 78 subjects each.

**3.2.1 Inclusion criteria**

- Medically fit
- Compliance: ablity to undergo examaination unassisted
- Tooth previously unrestored
- No pain
- Class I or Class II carious cavity in one or two primary molars teeth
- Absence of clinical a history and signs of pulp necrosis and infection
- Radiographic evidence caries prior to restorative treatment
  - 1. **Exclusion criteria**

Any of the following is regarded as a criterion for exclusion from the study:

- Demographic inaccessabilty
- Poor co-operation and inability to undergo clinical examination unaided
- Historic or ongoing pain
- Evidence of clinical pulp involvement: active sinus, abscess
- Evidence of radiographic pulp involvment
  - 1. **Justification for inclusion and exclusion criteria**

The criteria are set to minimize the risk to the volunteers, to ensure a subject population that will enable the investigation of the set objectives, to provide equal opportunity for inclusion and not bias the study with children whose behavior or understanding may influence the results.

- - 1. **Criteria for discontinuation**

Subjects may be discontinued from study treatment and assessments at any time, at the discretion of the investigator. Specific reasons for discontinuing a subject from the study are:

1. Withdrawal of informed consent.
2. Development of exclusion criteria, such as severe pain, abscess or attending another dentist and having treatment carried on the same tooth.
3. Protocol non-compliance.
4. Incorrect enrolment or randomization of the subject based on behavior during the treatment.

For subjects withdrawn from the study, the same measurements and assessments should be performed as done at Visit 5. Adverse events should be followed up and emergency treatment provided should need arise.

**3.3 Investigational Products and Treatments**

**3.3.1 Treatment Schedule**

At Visit 1, eligible subjects will receive the PMC as either by the HT or by the Conventional

Technique.

- - 1. **Randomization**

At Visit 1, subjects who are screened for the study will receive an enrolment code: H code for HT and C for conventional technique+ a consecutive number of 2 digits. Subjects who fulfill all inclusion criteria and meet none of the exclusion criteria will be allocated a subject number: S – code = S + a 2-digit number. The order of receiving the HT PMC and conventional technique PMCs will be in accordance with the code of randomization after tossing a coin. Eligible children, who for whatever reason, do not complete the course of treatment or who do not attend on review dates will be referred to as discontinuers in the report. Once allocated, the children randomization numbers will be used to identify them during the remainder of the study. A volunteer who, for whatever reason, withdraws or is withdrawn from the after having been allocated a subject randomization number and after the PMC was placed, will be classified as a dropout, and identified as such in the relevant Case Report Form (CRF).

**3.3.3 Identity of study products**

The study product is a standard disinfected preformed metal crowns, sizes 1-6 manufactured by Shinhung Co. Ltd, Korea.

**3.3.4 Storage and Accountability**

All study materials must be kept in a secure place under adequate storage conditions and from the designated box – protected from any contamination. Records of the PMC used will maintained in the patient’s records.

**3.3.5 Allowed restorative care and medicines**

Preferably no other restorative care for the tooth or any form of medicines such as analgesia or antibiotics should be administered to the subjects. The use of any incidental medication (e.g. mild analgesics, oral antibiotics, etc.) must be recorded.

- - 1. **Compliance**

The dentist will record adverse events and clinical outcomes and will be checked at every clinic visit.

##### STUDY MEASUREMENTS AND ENDPOINTS

#### In this study the following endpoints will be measured/recorded

- 1. **Primary Endpoints.**

1. Primary tooth survival
2. Adverse events (type and frequency)
   1. **Secondary Endpoints**
3. Anxiety
4. Gingival pathology measured by gingival index (Appendix 10.3, Table 3)
5. Oral hygiene as assesses by plaque index (Appendix 10.3, Table 2)
6. Occlusion
   1. **Measurements at each visit.**

See section 3.1.2 for the visit schedule.

**Visit 1: Screening and treatment visit**

The subjects; children aged between age 5-8 years, will be examined to assess their eligibility to participate. Each subject will consent in writing (Appendix 10.2) to the screening process before the start of the examination and treatment. The consent form will also include the study information leaflet.

The examinations and investigations will include:

- Medical and dental history, including history of past use of medications, demographics (date of birth, sex, race), parent job, insurance type.
- Physical examination including assessment of general appearance and compliance.
- Baseline radiograph, either dental pentamograph or bitewing radiographs if X-ray machines are available.
- Preoperative facial image scale questionnaire administered
- PMC fitted using HT or CT and treatment time noted
- Postoperative facial image scale questionnaire administered

**Visit 2-5: 6 months review.**

- Children will be seen at the Trial Center and investigator will confirm the tooth is functional and still eligible for inclusion in the study.
- Adverse effects on gingival health to be measured using gingival index (gingival hyperplasia and bleeding) (Appendix 10.3, Table 1) (4,5)
- Oral hygiene to be measured using plaque index (Appendix 10.3, Table 2) (4,5)
- Occlusal contacts will be examined; whether adjacent teeth were in contact or not.
- Facial image scale questionnaire will be administered to assess anxiety (Appendix 10.3, Figure 3) (3)

**Note:** For any subject with gingival problems, oral hygiene instructions will be reinforced, and future visits scheduled only if there is acute pathology that requires urgent intervention.

- 1. **Specific detail on measurements**

**4.4.1 Adverse events**

An adverse event is the development of an undesirable medical condition - e.g. abscess, gingival swelling, allergy, severe pain on biting or otherwise.

Mild = awareness of sign or symptom, but easily tolerated

Moderate = discomfort sufficient to cause interference with biting but not sleeping

Severe = incapacitating, with inability to perform normal oral function and interferes with eating and sleeping or the presence of a facial swelling.

A Serious Adverse Event is an adverse event occurring during any phase of the study and at any arm of the investigation and fulfills the following criteria:

- Results in abscess;
- Is immediately detrimental to health such as developing a fever or allergy;
- Requires pulp extrupation or tooth extraction;
- Results in persistent or significant dental incapacity.

The causality of Serious Adverse Events (i.e. the relationship to study treatment) will be assessed by the investigators, who in completing the relevant Case Report Form must answer ‘yes’ or ‘no’ to the question “Do you consider that there is a reasonable possibility that the event may have been caused by the PMC?” The following factors should be considered when deciding if there is a “reasonable possibility” that an Adverse Event may have been caused by the PMC rather than failure to accurately diagnose.

- Time course of events and exposure – did the AE occur in a reasonable temporal relationship to the time of treatment?
- Dechallenge experience – did the AE resolve or improve PMC removal, pulp treatment or tooth extraction?
- Rechallenge experience - did the AE reoccur if the PMC was adjusted and refitted?
- Laboratory tests – has a radiographs confirmed the relationship?
- No alternative cause - the AE cannot be reasonably explained by another aetiology such as an underlying disease (not previously present), misdiagnosis or progression of disease.

There would not be a “reasonable possibility” of causality if none of the above criteria apply or where there is evidence of disease progression and a reasonable time course, but any dechallenge is negative or there is another more likely cause of the AE.

In this study the Adverse Events will be noted from the interview at the time of visit and treatment decided accordingly.

- - 1. **Clinical examination**

A standard sterile dental mirror, porbe and tweezers and cotton pellets were used to examine carious teeth. No active excavation was undertaken.

- - 1. **Radiographic examination**

Blood and urine will be collected for clinical pathology tests at the screening visit, at visits 3 to 5, and, if required, at any post-study safety follow-up visits (or upon withdrawal). The following variables will be measured.

As part of the overall safety monitoring plan, the safety monitor (Dr F Elamin or Dr N Abdelazim) will also assess the data, particularly those teeth who are deemed to require invasive treatment.

1. **STATISTICAL METHODS**
   1. **Determination of sample size**

The sample size was calculated using PS Power and Sample size calculator Program; Version 3.1 using 80% power and α = 0.05 (two-sided), based on previously reported failure rates of 5% and 15% for both HT and CT SSCs (6,7). A sample size of 63 in each arm of the trial was needed to detect a significant difference. The minimum recruitment rate for each arm was 78 patients with the assumption that the drop out rate is 20%.

- 1. **Statistical analysis**

A single statistical analysis will be performed at the end of the study. An intention to treat (ITT) approach will followed, i.e. statistical analysis of safety will be based on data from all patients who were randomized and from whom meaningful data were collected. Data will be displayed graphically for visual inspection. Descriptive statistics will be presented as means, SEM and ninety percent confidence levels of the means.

**Baseline characteristics** Demographic, background and baseline data will be presented descriptively. Group means will be compared using student t test.

**Survival**. Survival will be reported on the presence or or absence of a PMC that can reasonably function Kaplan–Meier survival curvess and the log-rank test will be used to compare groups.

**Analysis of Safety.** Adverse Events, as reported throughout the course of the trial will be listed individually, per treatment group. Pre-, study and post-study findings of **anxiety (3), plaque and gingival (4,5) indcies and occlusion** will be compared.

- 1. **Changes to the Clinical Study Protocol**

The Local Ethics Committees (e.g. Independent Ethics Committee at KCRMT) will be notified of any amendments to the Clinical Study Protocol and no changes will be made without approval from the Regulatory Authorities.

1. **ETHICS**

**6.1 Ethics review**

The final study protocol, including the final version of the Subject Infrormation and Consent Forms, must be approved in writing by an Independent Ethics Committee (IEC) at KCRMT before enrolment of any subject into the study. The Principle Investigator (Clinical Trial Manager) is responsible for informing the IEC of any serious adverse events (SAE) and amendment to the protocol as per regulatory requirement.

**6.2. Ethical conduct of the study**

The study will be performed in accordance with the ethical principles in the Declaration of Helsinki (see Appendix 10.1), and that are consistent with Good Clinical Practice and applicable regulatory requirements. Insurance cover will be taken out by TICIPS to cover

- 1. **Subject information and consent**

The Investigator will ensure that the subject is given full and adequate oral and written information about the nature, purpose, possible risk and benefit of the study. Subjects must also be notified that they are free to discontinue from the study at any time. The subject should be given the opportunity to ask questions and allowed time to consider the information provided. The subject’s signed and dated informed consent must be obtained before conducting any study specific procedure. The investigator must store the original, signed Subject Informed Consent Form and a copy must be given to the subject. Samples of the English version of the Subject Information and Consent Forms are enclosed (Appendix 10.2). Arabic translations of the approved English version will also be provided and submitted to IEC.

- 1. **Subject data protection**

The Subject Information and Consent Form will explain that study data will be stored in a computer database, maintaining confidentiality. Subjects in this database will be identified by initials or enrolment code / subject number only. Authorized representative of a regulatory authority may require direct access to parts of the trial site records relevant to the study, including subjects’ medical history for data verification purposes.

The Investigator must keep a Subject Identification List of all subjects that have signed the informed consent.

**7. DATA QUALITY ASSURANCE**

Data from the study will be collected in CRFs. Data editing will be performed at the trial center, comparing source and CRF entries. Data will be entered in a blind mode.

During the study an independent monitor will visit the investigational site to confirm that the facilities remain acceptable, that the investigational team is adhering to the protocol and that data are being accurately recorded in the CRFs. Source data verification (a comparison of the data in the CRF with the subjects laboratory test results and other source documents) will also be performed.

Authorized representatives of the regulatory authority may visit the center to perform inspections, including source data verification.

Clean File for the final database will be declared when all data have been entered and a quality check on a sample of the data has been performed. The database will be locked after Clean File has been declared and data extracted for statistical analysis. Treatment code will not be broken until clean file.

Study committee meetings will be held as needed prior to or during the study. The medical, nursing and other staff involved in the study will receive proper education/information on how to conduct the study according to the protocol.

**8. STUDY TIME TABLE AND TERMINATION**

First subject in Feb 2015

Last patient out March 2016

Study Report September 2018

**9. REFERENCES**

1. Innes NPT, Stirrups DR, Evans DJP, Hall N, Leggate M. A novel technique using preformed metal crowns for managing carious primary molars in general practice—a retrospective analysis. Br Dent J. Nature Publishing Group; 2006;200: 451.
2. Kindelan SA, Day P, Nichol R, Willmott N, Fayle SA. UK National Clinical Guidelines in Paediatric Dentistry: stainless steel preformed crowns for primary molars. doi:10.1111/j.1365-263X.2008.00935.x
3. Buchanan H, Niven N. Validation of a Facial Image Scale to assess child dental anxiety. Int J Paediatr Dent. 2002;12: 47–52. doi:10.1046/j.0960-7439.2001.00317.x
4. Löe H. The gingival index, the plaque index and the retention index systems. J Periodontol. Am Acad Periodontology; 1967;38: 610–616.
5. 22. Löe H, Fehr FR, Schiött CR. Inhibition of experimental caries by plaque prevention. Eur J Oral Sci. Wiley Online Library; 1972;80: 1–9.
6. Innes NPT, Stirrups DR, Evans DJP, Hall N, Leggate M. A novel technique using preformed metal crowns for managing carious primary molars in general practice—a retrospective analysis. Br Dent J. Nature Publishing Group; 2006;200: 451.
7. 18. Santamaria RM, Innes NPT, Machiulskiene V, Evans DJP, Splieth CH. Caries management strategies for primary molars: 1-yr randomized control trial results. J Dent Res. 2014;93: 1062–9. doi:10.1177/0022034514550717

###### APPENDIX 10.1

DECLARATION OF HELSINKI

| **WORLD MEDICAL ASSOCIATION DECLARATION OF HELSINKI Ethical Principles for Medical Research Involving Human Subjects** |  |
| --- | --- |

Adopted by the 18th WMA General Assembly, Helsinki, Finland, June 1964, and amended by the
29th WMA General Assembly, Tokyo, Japan, October 1975
35th WMA General Assembly, Venice, Italy, October 1983
41st WMA General Assembly, Hong Kong, September 1989
48th WMA General Assembly, Somerset West, Republic of South Africa, October 1996
and the 52nd WMA General Assembly, Edinburgh, Scotland, October 2000
Note of Clarification on Paragraph 29 added by the WMA General Assembly, Washington 2002

**INTRODUCTION**

- 1. The World Medical Association has developed the Declaration of Helsinki as a statement of ethical principles to provide guidance to physicians and other participants in medical research involving human subjects. Medical research involving human subjects includes research on identifiable human material or identifiable data.
  2. It is the duty of the physician to promote and safeguard the health of the people. The physician's knowledge and conscience are dedicated to the fulfillment of this duty.
  3. The Declaration of Geneva of the World Medical Association binds the physician with the words, "The health of my patient will be my first consideration," and the International Code of Medical Ethics declares that, "A physician shall act only in the patient's interest when providing medical care which might have the effect of weakening the physical and mental condition of the patient."
  4. Medical progress is based on research, which ultimately must rest in part on experimentation involving human subjects.
  5. In medical research on human subjects, considerations related to the well-being of the human subject should take precedence over the interests of science and society.
  6. The primary purpose of medical research involving human subjects is to improve prophylactic, diagnostic and therapeutic procedures and the understanding of the aetiology and pathogenesis of disease. Even the best-proven prophylactic, diagnostic, and therapeutic methods must continuously be challenged through research for their effectiveness, efficiency, accessibility and quality.
  7. In current medical practice and in medical research, most prophylactic, diagnostic and therapeutic procedures involve risks and burdens.
  8. Medical research is subject to ethical standards that promote respect for all human beings and protect their health and rights. Some research populations are vulnerable and need special protection. The particular needs of the economically and medically disadvantaged must be recognized. Special attention is also required for those who cannot give or refuse consent for themselves, for those who may be subject to giving consent under duress, for those who will not benefit personally from the research and for those for whom the research is combined with care.
  9. Research Investigators should be aware of the ethical, legal and regulatory requirements for research on human subjects in their own countries as well as applicable international requirements. No national ethical, legal or regulatory requirement should be allowed to reduce or eliminate any of the protections for human subjects set forth in this Declaration.

1. **BASIC PRINCIPLES FOR ALL MEDICAL RESEARCH**
   1. It is the duty of the physician in medical research to protect the life, health, privacy, and dignity of the human subject.
   2. Medical research involving human subjects must conform to generally accepted scientific principles, be based on a thorough knowledge of the scientific literature, other relevant sources of information, and on adequate laboratory and, where appropriate, animal experimentation.
   3. Appropriate caution must be exercised in the conduct of research, which may affect the environment, and the welfare of animals used for research must be respected.
   4. The design and performance of each experimental procedure involving human subjects should be clearly formulated in an experimental protocol. This protocol should be submitted for consideration, comment, guidance, and where appropriate, approval to a specially appointed ethical review committee, which must be independent of the investigator, the sponsor or any other kind of undue influence. This independent committee should be in conformity with the laws and regulations of the country in which the research experiment is performed. The committee has the right to monitor ongoing trials. The researcher has the obligation to provide monitoring information to the committee, especially any serious adverse events. The researcher should also submit to the committee, for review, information regarding funding, sponsors, institutional affiliations, other potential conflicts of interest and incentives for subjects.
   5. The research protocol should always contain a statement of the ethical considerations involved and should indicate that there is compliance with the principles enunciated in this Declaration.
   6. Medical research involving human subjects should be conducted only by scientifically qualified persons and under the supervision of a clinically competent medical person. The responsibility for the human subject must always rest with a medically qualified person and never rest on the subject of the research, even though the subject has given consent.
   7. Every medical research project involving human subjects should be preceded by careful assessment of predictable risks and burdens in comparison with foreseeable benefits to the subject or to others. This does not preclude the participation of healthy volunteers in medical research. The design of all studies should be publicly available.
   8. Physicians should abstain from engaging in research projects involving human subjects unless they are confident that the risks involved have been adequately assessed and can be satisfactorily managed. Physicians should cease any investigation if the risks are found to outweigh the potential benefits or if there is conclusive proof of positive and beneficial results.
   9. Medical research involving human subjects should only be conducted if the importance of the objective outweighs the inherent risks and burdens to the subject. This is especially important when the human subjects are healthy volunteers.
   10. Medical research is only justified if there is a reasonable likelihood that the populations in which the research is carried out stand to benefit from the results of the research.
   11. The subjects must be volunteers and informed participants in the research project.
   12. The right of research subjects to safeguard their integrity must always be respected. Every precaution should be taken to respect the privacy of the subject, the confidentiality of the patient's information and to minimize the impact of the study on the subject's physical and mental integrity and on the personality of the subject.
   13. In any research on human beings, each potential subject must be adequately informed of the aims, methods, sources of funding, any possible conflicts of interest, institutional affiliations of the researcher, the anticipated benefits and potential risks of the study and the discomfort it may entail. The subject should be informed of the right to abstain from participation in the study or to withdraw consent to participate at any time without reprisal. After ensuring that the subject has understood the information, the physician should then obtain the subject's freely given informed consent, preferably in writing. If the consent cannot be obtained in writing, the non-written consent must be formally documented and witnessed.
   14. When obtaining informed consent for the research project the physician should be particularly cautious if the subject is in a dependent relationship with the physician or may consent under duress. In that case the informed consent should be obtained by a well-informed physician who is not engaged in the investigation and who is completely independent of this relationship.
   15. For a research subject who is legally incompetent, physically or mentally incapable of giving consent or is a legally incompetent minor, the investigator must obtain informed consent from the legally authorized representative in accordance with applicable law. These groups should not be included in research unless the research is necessary to promote the health of the population represented and this research cannot instead be performed on legally competent persons.
   16. When a subject deemed legally incompetent, such as a minor child, is able to give assent to decisions about participation in research, the investigator must obtain that assent in addition to the consent of the legally authorized representative.
   17. Research on individuals from whom it is not possible to obtain consent, including proxy or advance consent, should be done only if the physical/mental condition that prevents obtaining informed consent is a necessary characteristic of the research population. The specific reasons for involving research subjects with a condition that renders them unable to give informed consent should be stated in the experimental protocol for consideration and approval of the review committee. The protocol should state that consent to remain in the research should be obtained as soon as possible from the individual or a legally authorized surrogate.
   18. Both authors and publishers have ethical obligations. In publication of the results of research, the investigators are obliged to preserve the accuracy of the results. Negative as well as positive results should be published or otherwise publicly available. Sources of funding, institutional affiliations and any possible conflicts of interest should be declared in the publication. Reports of experimentation not in accordance with the principles laid down in this Declaration should not be accepted for publication.
2. **ADDITIONAL PRINCIPLES FOR MEDICAL RESEARCH COMBINED WITH MEDICAL CARE**
   1. The physician may combine medical research with medical care, only to the extent that the research is justified by its potential prophylactic, diagnostic or therapeutic value. When medical research is combined with medical care, additional standards apply to protect the patients who are research subjects.
   2. The benefits, risks, burdens and effectiveness of a new method should be tested against those of the best current prophylactic, diagnostic, and therapeutic methods. This does not exclude the use of placebo, or no treatment, in studies where no proven prophylactic, diagnostic or therapeutic method exists. See footnote
   3. At the conclusion of the study, every patient entered into the study should be assured of access to the best-proven prophylactic, diagnostic and therapeutic methods identified by the study.
   4. The physician should fully inform the patient which aspects of the care are related to the research. The refusal of a patient to participate in a study must never interfere with the patient-physician relationship.
   5. In the treatment of a patient, where proven prophylactic, diagnostic and therapeutic methods do not exist or have been ineffective, the physician, with informed consent from the patient, must be free to use unproven or new prophylactic, diagnostic and therapeutic measures, if in the physician's judgement it offers hope of saving life, re-establishing health or alleviating suffering. Where possible, these measures should be made the object of research, designed to evaluate their safety and efficacy. In all cases, new information should be recorded and, where appropriate, published. The other relevant guidelines of this Declaration should be followed.

**FOOTNOTE:
NOTE OF CLARIFICATION ON PARAGRAPH 29 of the WMA DECLARATION OF HELSINKI**

The WMA hereby reaffirms its position that extreme care must be taken in making use of a placebo-controlled trial and that in general this methodology should only be used in the absence of existing proven therapy. However, a placebo-controlled trial may be ethically acceptable, even if proven therapy is available, under the following circumstances:

- Where for compelling and scientifically sound methodological reasons its use is necessary to determine the efficacy or safety of a prophylactic, diagnostic or therapeutic method; or

- Where a prophylactic, diagnostic or therapeutic method is being investigated for a minor condition and the patients who receive placebo will not be subject to any additional risk of serious or irreversible harm.

All other provisions of the Declaration of Helsinki must be adhered to, especially the need for appropriate ethical and scientific review.

The Declaration of Helsinki (Document 17.C) is an official policy document of the World Medical Association, the global representative body for physicians. It was first adopted in 1964 (Helsinki, Finland) and revised in 1975 (Tokyo, Japan), 1983 (Venice, Italy), 1989 (Hong Kong), 1996 (Somerset-West, South Africa) and 2000 (Edinburgh, Scotland). Note of clarification on Paragraph 29 added by the WMA General Assembly, Washington 2002.

**APPENDIX 10.2**

**PATIENT INFORMATION LEAFLET AND INFORMED CONSENT**

PATIENT INFORMATION SHEET AND

INFORMED CONSENT FORM

**Patient identification number: . . . . / . . . . . Patient initials:**

**Title of the study:** A randomized clinical trial comparing Hall and conventional preformed metal crown placement techniques from Sudan.

**Protocol number:** Paed/Dent/11-01

**Name of Principal Investigator:** Dr Fadil Elamin

**Name of Project Leader:** Prof FSL Wong

**Address of site/institution where study will be conducted:** Dental Centre,, 4rth Floor, Islamic Bank building, Khartoum, Sudan

**INTRODUCTION**

You are being invited to take part in a clinical research study to assess to forms of reconized dental treatments that are routinely used to treat decayed teeth in children in many parts of the world. Before deciding to take part in the study, it is important for you to understand why the research is done and what will happen to you. This information sheet will provide you with information about this study and your rights as a research subject so that you can decide if you want to take part. Please take the time to read this information carefully and ask the investigator when anything is not clear, or if you would like more information*.*

What is the purpose of this study?

The purpose of the study is to establish how a new techniques of partially removing caries and placing a stainless steel crown with miminal intervention and with no local anaesthetic compares to the more conventional treatment which requires the denist to administer local anaesthtetc remove all the decayed part of the tooth and place the stainless steel crown.

**Do I have to participate in the study?**

Participation of your child in this study is entirely voluntary. It is up to you to decide whether to take part or not.

If you do not want to take part in this study or if you wish to withdraw from the study at any time you may do so without giving a reason and you will not lose any benefits to which you would otherwise be entitled and your treatment will not be affected.

If you withdraw from the study the data collected up to the point of withdrawal will be analyzed for the purpose of the study.

If you decide to participate, you will be given this information sheet to keep and be asked to provide your signature indicating your consent.

**Who is eligible to participate in the study?**

About 160 children (older than 5 years and younger than 8) will take part in this study. In order to participate in this trial your child needs to be medically healthy and cooperative. They have to have decayed teeth that can easily be treated and does not require extensitve root therapy.

There is not enough information currently on which technique is more beneficial for your child and all treatments are designed to deal with the initial problem your child presents with in a way that will be acceptable to other dentists.

**What will happen to me if I participate?**

If you decide to take part you must sign the consent form and will then first be examined to see if you meet all the requirements for participation in the study. These examinations and investigations (at visit 1) will include the taking of a medical history history, a radiograph and help your child fill a simple smiley questionnaire before and after the treatment provided.

**What do I have to do?**

As a subject/parent in this study, you are responsible for:

- keeping all clinic appointments;
- Reporting to us if you think your child is having any problems

**What risks or discomforts might occur if I participate?**

The child may experience pain on biting on any new restoration and includes newly fitted crowns. As with any dental treatment complications such pulp death may follow and will require you to tooth to be root treated or even extracted.

Many of the procedures to be used in this study may cause you some discomfort but the dentists and therapists are trained to make sure the child is as comfortable as possible using safe recongnized procedures and will make sure the child is under control.

**What are the possible benefits of participating?**

You may or may not receive any direct benefit from taking the study medication, but all the regular visits and examinations to monitor your health will be provided immedialtely without having to wait for hours to receive care. Further, the information we get from this study will extend our existing knowledge and will help to decide if this treatment is effective and can be used on other people such as you.

**Will there be any cost to me if I participate?**

There will be no cost to you for participating in this research study, but you will need to allow enough time for the clinic visits.

**What if something goes wrong or if I have problems while I am in the study?**

In case of study related problems please contact:

Doctor: Dr Nihal Abd Elazim; Medical Director

Contact Numbers: 0024922805635

**Can I withdraw or be withdrawn from the study?**

Taking part in the study is voluntary. If you decide to take part in the study, you are free to withdraw from the study at any time. If you decide to withdraw from the study, you should inform your study dentist immediately. Your study dentist will not be upset and you will not be penalized in any way, and your future care will not be affected. Should you withdraw from the study, the study data collected before your withdrawal may still be processed along with other data collected as part of the study.

In addition, circumstances may arise that will lead to the ending of your participation in this study. Such circumstances could include:

- medical reasons;
- if you do not attend review appointments if there are not enough patients in the study;
- if we have to carry extensive treatment on the tooth we already treated.
- If we, or our organizing ethical committee, stop the study.

**Will my participation in this study be kept confidential?**

The records that identify you will be kept confidential and, to the extent permitted by the applicable laws and regulations, will not be made publicly available.

The information collected during the study will be stored in a computer but your name will not be stored. Only your study doctor will know that the information is related to you. All blood collected during the study will be labeled with your anonymous subject number and initials. Samples will be sent to a central laboratory for analysis in batches, but your name will not be included on any specimens or accompanying documentation that is sent to the laboratory.

The results of the study may be published in the medical literature and/or presented at a scientific conference or symposium, but your identity will not be revealed. The information disclosed will be collective summarized data.

You may ask to see your medical information as prescribed by law. The treatment that you received in the study needs to remain unknown (blinded) until the study data is analyzed; you may see this information, but only after the data has been analyzed.

**Who has reviewed the study?**

This study has been approved by the ethical committee of the the centre and this is in accordance with the guidelines of the International Conference on Harmonization (ICH) for Good Clinical Practice (GCP) and with the Declaration of Helsinki (version 2000). These are policy statements that protect the rights of study patients and volunteers.

If you require details about these committees, you should ask the study dentist (or project leader; Dr Fadil Elamin).

321

**Contacts for further information.**

If during the course of this study, you have questions about the nature of the research or your rights, or you believe that you have sustained a research-related injury, you should contact one of the following:

Dr Fadil Elamin (study doctor) at 00249922805635

Dr Nihal Abdelazim (medical director 00249922805635

If you have any questions about your rights as a participant in a research experiment/trial, you can contact the Committee for Pharmaceutical Trials of the University of Stellenbosch at (021) 938 9075.

If neither the study doctor nor the independent Ethics Committee can assist you, you may contact:

# Dr Yoursra Mirghani

College of Medicine

University of Khartoum

Al Qasr Streeth

Khartoum

Sudan

INFORMED CONSENT FORM

**Patient identification number: . . . . / . . . . . Patient initials:**

**Project title:** A randomized clinical trial comparing Hall and conventional preformed metal crown placement techniques from Sudan.

Protocol number: Paed/Dent/11-01

By signing and dating this document,

- I confirm that I have had time to carefully read and understand the patient information sheet provided for this study.
- I confirm that I have had the opportunity to discuss the study and ask questions and I am satisfied with the answers and explanations that I have been provided.
- I understand that my participation is voluntary and that I am free to withdraw at any time without giving any reason and without my medical care or legal rights being affected.
- I confirm that I have received a signed and dated copy of the Patient Information Sheet and Informed Consent Forms.

Subject: _________________ _______________ ______________

Name (capital letters) Date

*Parent/Garudian: _________________ _______________ _________________*

*(required)*

Name (capital letters) Signature Date

*PERSON OBTAINING CONSENT*:

_________________ _______________ ________________

Name (capital letters) Signature Date

*CLINICAL INVESTIGATOR*:

_________________ _______________ ________________

Name (capital letters) Signature Date

**توضيح للمريض**

معلومات للمريض

استمارة الموافقة المسبقة

**الرقم التعريفي للمريض**

**عنوان الدراسة : تجربة سريرية عشوائية للمقارنة بين الطريقة التقليدية لتلبيسات الاطفال وطريقة هول من السودان**

**رقم البروتوكول:**Paed/dent/11-01

**اسم الباحث الرئيسي : د/ فاضل الامين**

**Prof FSL Wong** **اسم مدير المشوع /**

**عنوان مكان الدراسة**

**الخرطوم –السودان –عمارة البنك الاسلامي الاسلامي –مركز طب الاسنان الطابق الرابع**

**المقدمة**

**ندعوك للمشاركة في البحث السريري لدراسة طريقة روتيتنية ومتعارف عليه عالما لعلاج تسوسات اسنان الاطفال .**

**من المهم اعلامك باهمية البحث وماسيحدث اثناء البحث .ولك حرية المشاركة فية .**

**الرجاء اخذ الوقت والمعلومات الكافية من الباحث . اذا وجد اي معلومات عير واضحة .**

**اهداف البحث :-**

**دراسة طريقة جديدة لازالة التسوس ووضع التلبيسات المعدنية لاسنان الاطفال مقانة ب الطريقة التقليدية لوضح التلبيسات بالاستخدام البنج الموضعي وازالة الاجزاء المتضررة بالتسوس من السن**

**هل علي المشاركة في البحث :**

**المشاركة اختيارية .**

**في حال المشاركة للبحث يمكنك عدم الاكمال فية في حال قررت ذلك في اي وقت وليس عليك اعطاء اي اسباب .**

**في حال عدم الاكمال في البحث تعتبر المعلومات السابقة جزء من البحث وقابل للتحليل**

**في حال المشاركة في البحث تم اعطاك هذه المعلومات واقرارك بالموافقة عليها .**

**المشاركون في البحث**

**160 طفل من عمر 5 سنوات الي 8 سنوات . طفل متعاون ولا يعاني من مشاكل طبية**

**تسوسات سطحية لاتحتاج الي معالجة العصب**

**ماذا يحدث اذا كنت جزء من البحث**

**التوقيع علي الاقرار من الزيارة الاولي واخذ التاريخ الطبي الخضوع للاشعة مساعدة الطفل علي الاجابة علي بعض الاسئلة قبل وبعد العلاج**

**ما يترتب علي المشاركة في البحث**

**الحضور في لجميع المواعيد المقررة للعلاج**

**تبليغ الباحث في حال حدوث اي مشاكل للطفل**

**ماهي المشاكل الاخطار المترتبة في حال المشاركة في البحث**

**قد يعاني الطفل من بعض الالم عند المضغ بسبب الحشوات أو التلبيسات الجديدة كجزء طبيعي من علاج الاسنان .**

**ماهية فوائد المشاركة في البخث**

**لاتوجد فوائد مباشرة سوي مراقبة وعلاج اسنان الطفل من دون انتظار طويلة اثناء جلسات العلاج**

**هل علي اي تكاليف مالية في حال المشاركة في البحث :**

**لاتوجد اي تكاليف مالية**

**ماذا علية فعلة في حال حدوث اي مشاكل اثناء البحث**

**عليك الاتصال ب د/نهال عبد العظيم سيد احمد . المدير الطبي .**

**رقم الاتصال 0024922805635**

**هل المشاركة في هذا البخث سرية**

**كل المعلومات الماخذه من المريضة تبقي سرية ولن يتم نشرها للعامة .**

**كل المعلومات الماخذه في هذا البحث سيتم حفظها في جهاز كمبيوتر خاص لايتم كتابة اسم المريض فية**

**من قام بمراجعة هذا البحث**

**الجمعية العلمية لحقوق البحوث .**

**معلومات الاتصال**

**د/ فاضل الامين ( الطبيب الباحث )00249922805635**

**د/ نهال عبد العظيم ( المدير الطبي )00249922805635**

**استمارة الموافقة المسبقة**

**الرقم التعربفى:**

**عنوان الدراسة : تجربة سريرية عشوائية للمقارنة بين الطريقة التقليدية لتلبيسات الاطفال وطريقة هول من السودان**

**رقم البروتوكول:**Paed/dent/11-01

**من خلال التوقيع وتعود هذه الوثيقة،**

**أؤكد بأنني لديه وقت لقراءة بعناية وفهم ورقة معلومات المريض المقدمة لهذه الدراسة.**

**وأؤكد أن أتيحت لي الفرصة لمناقشة الدراسة وطرح الأسئلة، وأنا راض عن الإجابات والتفسيرات التي قدمت لي.**

**أنا أفهم أن مشاركتي طوعية وأنني حر في الانسحاب في أي وقت دون إبداء أي سبب ودون بلادي الرعاية الطبية أو الحقوق**

**القانونية التي تتأثر.**

**أؤكد أنني قد تلقيت موقعة ومؤرخة نسخة من المرضى ورقة معلومات ومطلعة أشكال الموافقة.**

**موضوع: _________________ _______________ ______________**

**اسم (حروف) تاريخ**

**الوالد / Garudian: _________________ _______________ _________________**

**(مطلوب)**

**اسم (حروف) تاريخ التوقيع**

**الشخص موافقة الحصول على:**

**_________________ _______________ ________________**

**اسم (حروف) التوقيع تاريخ**

**محقق السريرية:**

**_________________ _______________ ________________**

**اسم (حروف) التوقيع تاريخ**

###### APPENDIX 10.3

**Clinical Outcome Criteria**

- - 1. **Gingival Index (4)**


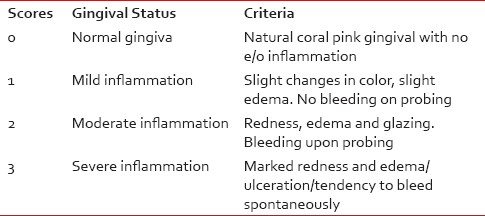


- - 1. **Plaque index (4,5)**


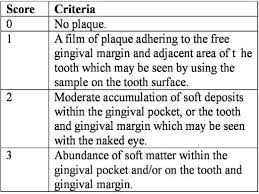


##### Facial Image Scale (3)


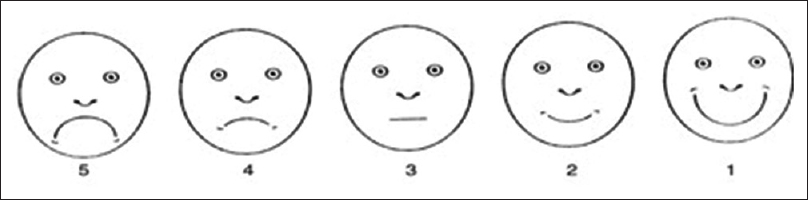

Supplement: S2 File — (DOCX) [file pone.0217740.s002.docx]
